# Supplementary material for: Probing and manipulating the Mexican hat-shaped valence band of In2Se3
Source: Nat Commun. 2025 Jan 22;16:922. doi: 10.1038/s41467-025-56139-8 (PMC11754478; doi:10.1038/s41467-025-56139-8)
Supplement: Supplementary file 1 — Supplementary Information [file 41467_2025_56139_MOESM1_ESM.pdf]

Supplementary Information

Probing and Manipulating the Mexican Hat-Shaped Valence Band of In_2Se_3

James Felton[1], Jordan Harknett[2], Joe Page[2], Zhuo Yang[3], Nada Alghofaili[1], James N. O'Shea[1], Laurence Eaves[1], Yoshimitsu Kohama[3], Mark T. Greenaway[2], and Amalia Patanè[1*]*

[1] School of Physics and Astronomy, University of Nottingham, Nottingham, NG7 2RD, UK

[2] Department of Physics, Loughborough University, Loughborough, LE11 3TU, UK

[3] Institute for Solid State Physics, The University of Tokyo, Kashiwa, Chiba, 277-8581, Japan

*Author to whom any correspondence should be addressed.

E-mail : amalia.patane@nottingham.ac.uk, m.t.greenaway@lboro.ac.uk

Table of contents

Supplementary Note 1. Atomic Force Microscopy

Supplementary Note 2. Raman spectroscopy

Supplementary Note 3. DFT for bulk 2H-α-In_2Se_3

Supplementary Note 4. ARPES for bulk 2H-α-In_2Se_3

Supplementary Note 5. Temperature dependence of the energy absorption edge for bulk 2H-α-In_2Se_3

Supplementary Note 6. Thermal annealing in UHV of bulk 2H-α-In_2Se_3

Supplementary Note 7. DFT for bulk α-In_2Se_3 and β-In_2Se_3

Supplementary Note 8. ARPES for bulk β-In_2Se_3

Supplementary Note 9. DFT for single layer α-In_2Se_3 and β-In_2Se_3

Supplementary Note 1. Atomic Force Microscopy

Atomic force microscopy (AFM) was used to assess the thickness of the In_2Se_3 flakes measured by nanoARPES. For both α and β phases of In_2Se_3, AFM was conducted in air, following the nanoARPES experiments and without any further annealing. The thickness of both In_2Se_3 flakes (0.3 μm and 0.4 μm for α and β phases, respectively) is above the threshold thickness at which bulk like behaviour is observed. Supplementary Figure 1 shows the AFM images of two flakes. The variation in flake thickness is of the order of 5 nm, equivalent to 6 van der Waals layers. The surface root mean square roughness values of 1.1 nm and 1.2 nm were measured for the α and β phases, respectively.

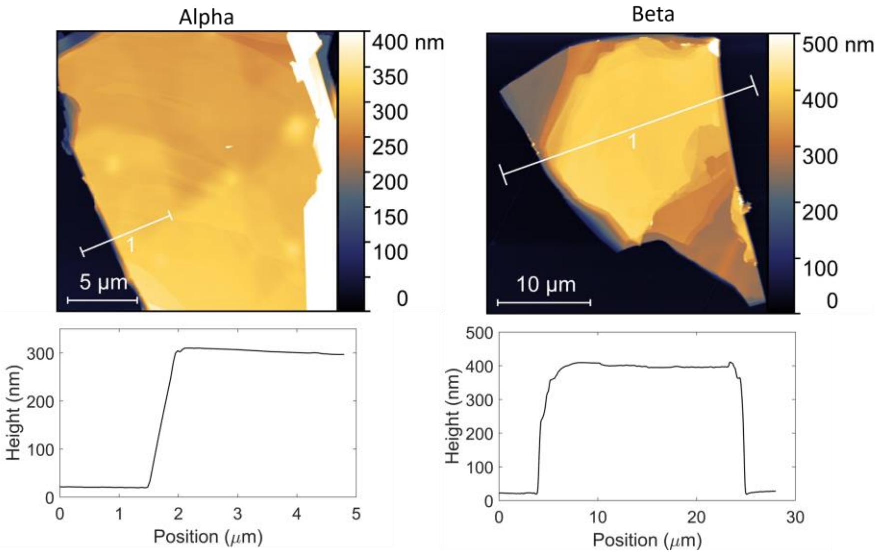

Supplementary Figure 1. Atomic Force Microscopy
AFM images of α- and β-In_2Se_3 flakes after the nanoARPES measurements. Shown beneath the maps are the height profiles of each flake along the lines shown in the images. AFM measurements were conducted in air at room temperature.

Supplementary Note 2. Raman spectroscopy

Samples were investigated by Raman spectroscopy in air. Focusing on the same locations used to gather the nanoARPES data, Raman spectra were obtained for laser spots of about 1 μm in diameter (λ = 632.8 nm and T = 300K). The typical Raman spectra are shown in Supplementary Figure 2. Five prominent peaks are seen in the spectrum for the α-phase of In_2Se_3. These are centred at 87.8 cm^{-1}, 103.3 cm^{-1}, 155.6 cm^{-1}, 179.6 cm^{-1}, and 185.1 cm^{-1}. In contrast, three main peaks were observed in the β phase, centred at 106.2 cm^{-1}, 171.6 cm^{-1}, and 202.8 cm^{-1}. For both samples, the peak positions are in good agreement with the values in the literature[1,2]. The strength of the Raman signal for the β-phase is similar to that for the α-phase. However, for the former the Raman peaks are broader, indicating a greater defectiveness in β-In_2Se_3.

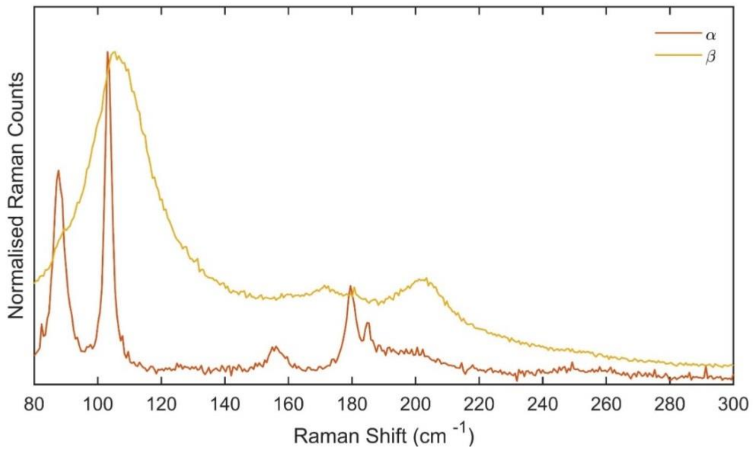

Supplementary Figure 2. Raman spectra
Raman spectra of α- and β-In_2Se_3 (λ=632.8 nm, T= 293 K).

Supplementary Note 3. DFT for bulk 2H-α-In_2Se_3

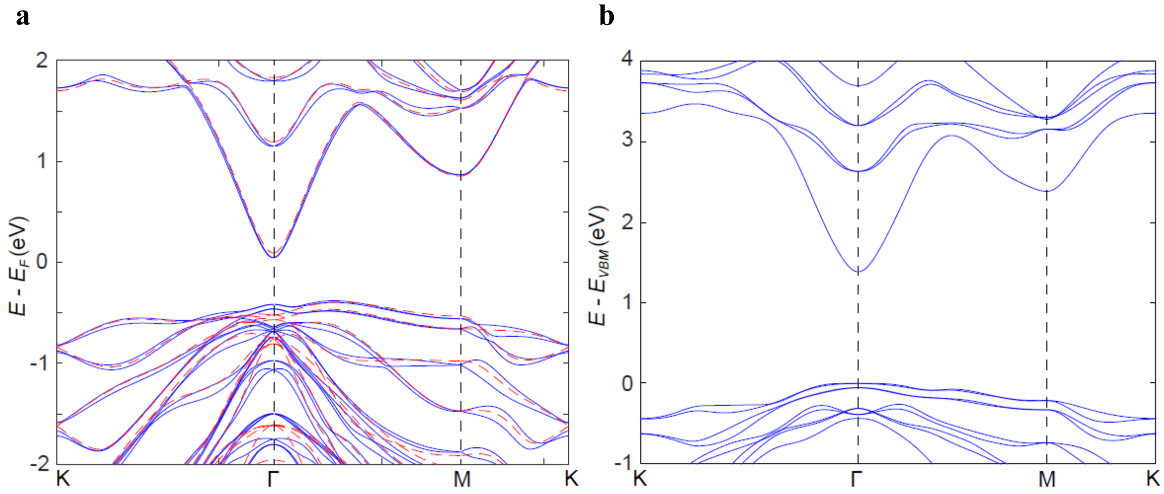

Supplementary Figure 3. Band structure for bulk 2H-α-In_2Se_3
(a) Band structure for bulk 2H-α-In_2Se_3 as determined by DFT with the PBE-GGA approximation of the exchange-correlation functional. Blue curves include SOC, red dashed curves are without SOC. (b) Band structure for bulk 2H-α-In_2Se_3 using the GW approximation with SOC.

Supplementary Figure 3a shows a comparison of the band structure of In_2Se_3 calculated using DFT and norm conserving pseudopotentials with (blue curves) and without (red dashed curves) spin orbit coupling. We find that in both cases, In_2Se_3 is an indirect semiconductor, with the valence band maximum located approximately 1/3 the distance between Γ and M. Our calculations reveal a small spin-orbit splitting of the bands, which is most significant between Γ and K (where it has a maximum of ~ 100 meV). In addition, we find that at Γ, coupling between the bands due to spin orbit interaction increases the energy of the upper-most valence

band at the Γ point relative to the valence band maximum, reducing the depth of the band inversion. As expected, the initial density functional theory calculations (with the PBE-GGA approximation of the exchange-correlation functional) significantly underestimates the band gap by ~1 eV. Therefore, in order to obtain a better estimate of the band-gap we used the GW approximation as implemented in the YAMBO package. We find that the GW correction increases the band gap to 1.38 eV (Supplementary Figure 3b), which is in much better agreement with the value determined experimentally from the optical transmission spectra. However, we also find that our calculation gives rise to flatter bands at the Γ point, which is in contrast to the experimentally determined values. The mass of the hole at the Γ point is -2.59 m_e compared to the experimentally determined value of (-0.87 ± 0.17) m_e. It is interesting to note that when we repeat the calculation without spin orbit interaction, the mass is reduced to -0.66 m_e. Spin orbit coupling and the GW approximation both tend to flatten the bands at the Γ point, showing the importance of considering these effects when determining the form of the valence band in materials with band inversion. This is particularly important in materials with a smaller band gap as the effects of the GW approximation may have a larger effect on the form of the valence bands.

Supplementary Figure 4 shows a colour plot of the electron energy versus in-plane *k*-vector, as determined by DFT using the GW approximation with SOC for the out-of-plane wave vector k_z at the edge of the BZ. In the supplementary movie, we show colour plots for different k_z.

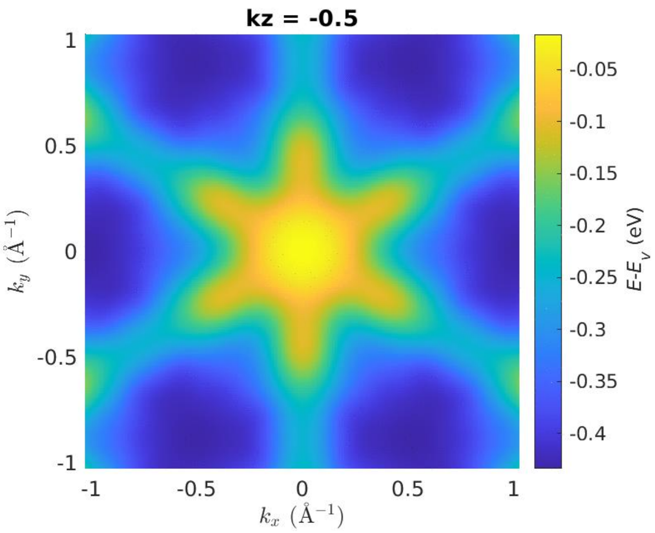

Supplementary Figure 4. Valence band for the out-of-plane wave vectors k_z > 0
Colour plot of the electron energy (in eV) versus in-plane *k*-vector, as determined by DFT using the GW approximation with SOC. The DFT plot corresponds to the out-of-plane wave vectors k_z > 0 at the edge of the BZ. See the supplementary movie for colour plots at different k_z.

Supplementary Note 4. ARPES for bulk 2H-α-In_2Se_3

The energy (E) vs k-vector dependence of the uppermost valence band was obtained from the raw ARPES intensity data by fitting the position of the photoemission onset and mapping its k-dependence. The profiles along the high symmetry $\bar{\Gamma}\rightarrow\bar{K}$ and $\bar{\Gamma}\rightarrow\bar{M}$ directions were then extracted. Parabolic fits to the energy dispersions at various points of the Brillouin zone were used to estimate the hole effective masses. This is shown for the 2H-α phase of In_2Se_3 in Supplementary Figure 5. The associated carrier effective masses are in Table 1 of the manuscript.

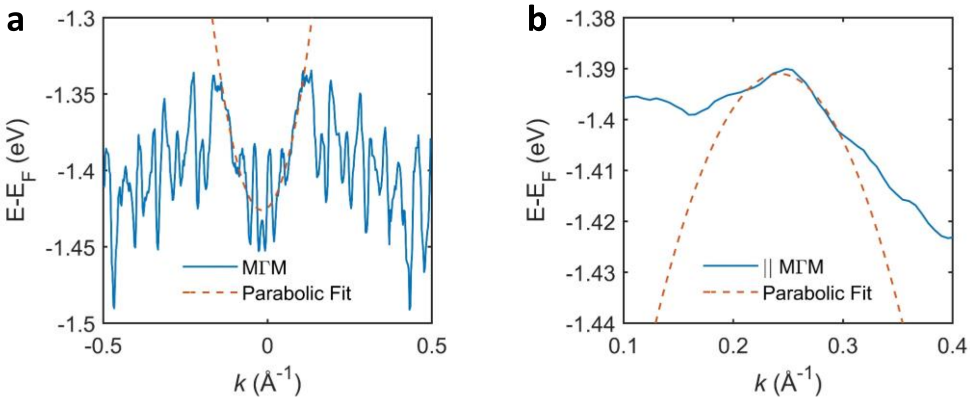

Supplementary Figure 5. Parabolic fits to the energy dispersions
(a) Parabolic fit (dashed lines) to the measured valence band of 2H-α-In_2Se_3 along the $\bar{\Gamma}\rightarrow\bar{M}$ cut around $k = 0$. (b) Parabolic fit at the VBM ($k > 0$) along the $\bar{\Gamma}\rightarrow\bar{M}$ cut. The derived hole effective masses are shown in Table 1 of the manuscript.

Supplementary Note 5. Temperature dependence of the energy absorption edge for 2H-α-In_2Se_3

The temperature dependence of the optical absorption edge of 2H-α-In_2Se_3 in zero magnetic field is shown in Figure 3b of the main manuscript. The position of this edge can be described by the simple O'Donnell-Chen model[3]

$$E_g(T) = E_g(0) - S\langle\hbar\omega\rangle[\coth(\langle\hbar\omega\rangle/2k_BT) - 1], \quad [1]$$

where $E_g(T)$ is the temperature-dependent band gap energy, $S = 6.3$ is a dimensionless constant, $\langle\hbar\omega\rangle = 23.3$ meV is the average phonon energy, and k_B is Boltzmann's constant. The resulting curve is plotted in the inset of Figure 3b.

Supplementary Note 6. Thermal annealing in UHV of bulk 2H-α-In_2Se_3

Annealing of 2H-α-In_2Se_3 was conducted in a stepwise manner, varying the annealing temperature T_a and duration. After each annealing, changes to the sample were monitored with energy-filtered photoemission electron microscopy (PEEM, Supplementary Figure 6a) and ultraviolet photoelectron spectroscopy (UPS, Supplementary Figure 6b) at room temperature.

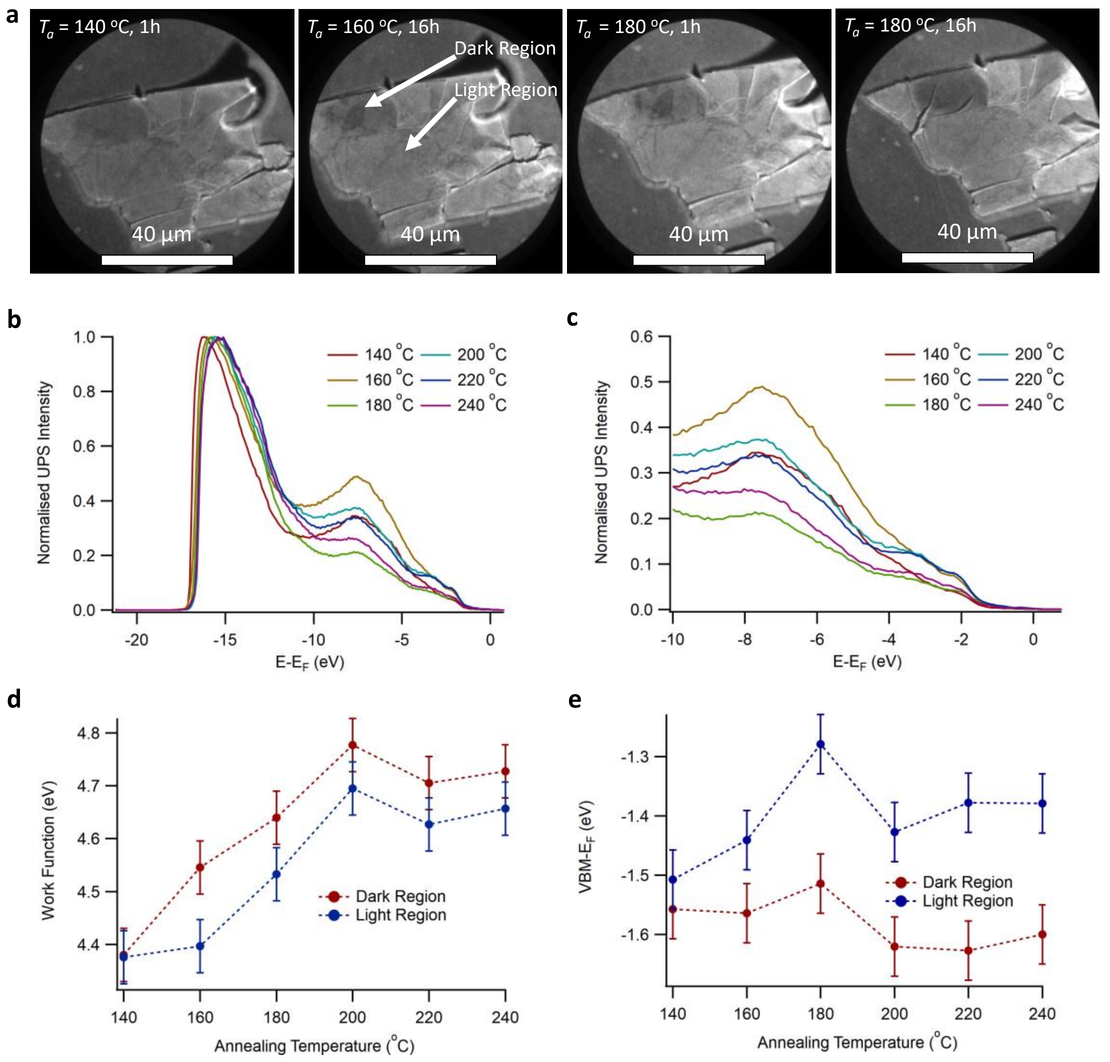

Supplementary Figure 6. Photoemission electron microscopy
a) PEEM images of 2H-α-In_2Se_3 after annealing at different temperatures. Arrows indicate regions of different colour whose work functions and VBM are compared in parts (d) and (e). b) UPS spectrum of In_2Se_3 after annealing at different temperatures. c) UPS spectrum in proximity of the VB region. d) Work function after each thermal annealing. e) Energy for the VBM after each thermal annealing.

The PEEM images reveal an inhomogeneous change across an In_2Se_3 flake with dark and bright regions, reflecting a localised change in the work function. Plots of the work function versus T_a shows an increase of up to ~ 0.3 eV across the flake, with slightly larger increases in the dark

regions of the PEEM images. A possible reason for this inhomogeneity is the emergence of wrinkles on the flake surface. This may point to strain playing a role in the observed changes. We cannot draw conclusions from the UPS data about changes induced by the annealing, except for the secondary electron cutoff, which can be explained by an increasing work function. Otherwise, there is no discernible trend in the position of the VBM.

Supplementary Note 7. DFT for bulk α-In_2Se_3 and β-In_2Se_3

Supplementary Figure 7 shows the DFT calculated band structure, including the GW approximation, of the 2H-α, 1T-β, 2H-β, and 3R-β phases of In_2Se_3. In transitioning from the α to β phase of In_2Se_3, the CBM moves away from the Γ point to the M point for the 1T and 2H stackings and towards the L point in the 3R stacking. There remains a local minimum at the Γ point. All β-stackings retain an IMH-shaped valence band. The degree of anisotropy also varies significantly between the stackings, being greatest in the 1T phase.

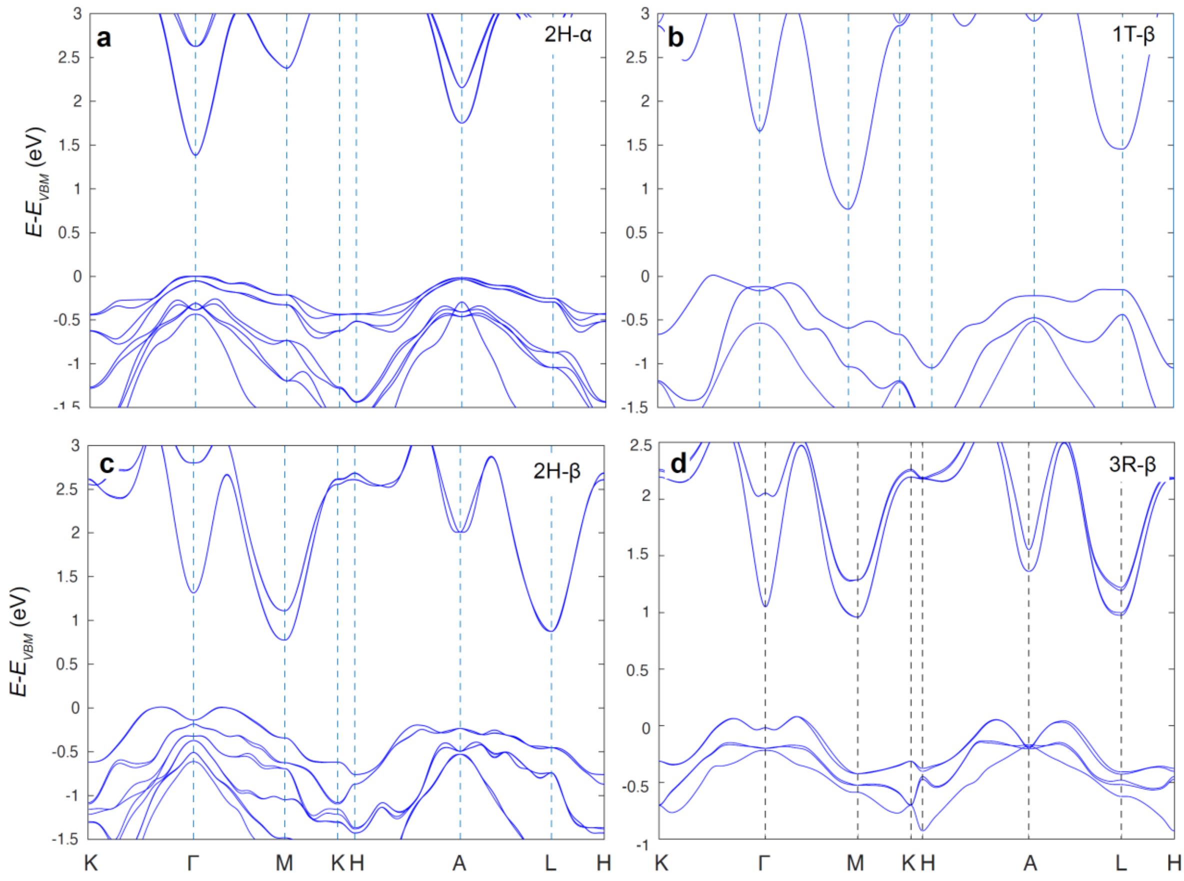

Supplementary Figure 7. Band structure for bulk α-In_2Se_3 and β-In_2Se_3
Calculated band structure for (a) 2H-α-In_2Se_3, (b) 1T-β-In_2Se_3, (c) 2H-β-In_2Se_3, and (d) 3R-β-In_2Se_3. The GW calculations include spin orbit coupling (SOC).

Supplementary Note 8. ARPES for bulk β-In_2Se_3

Parabolic fits to the measured ARPES energy dispersions of β-In_2Se_3 can yield an estimate of the carrier effective masses at key points in the Brillouin zone (Supplementary Figure 8). At the $\bar{\Gamma}$ point, this yields carrier effective masses of (-4.3±0.6)m_e and (-2.8±0.2)m_e along the $\bar{\Gamma} \rightarrow \bar{K}$ and $\bar{\Gamma} \rightarrow \bar{M}$ directions, respectively. At the VBM, the carrier effective mass is (4.0 ± 0.2)m_e and (2.6 ± 0.2)m_e along the $\bar{\Gamma} \rightarrow \bar{K}$ and $\bar{\Gamma} \rightarrow \bar{M}$ directions, respectively.

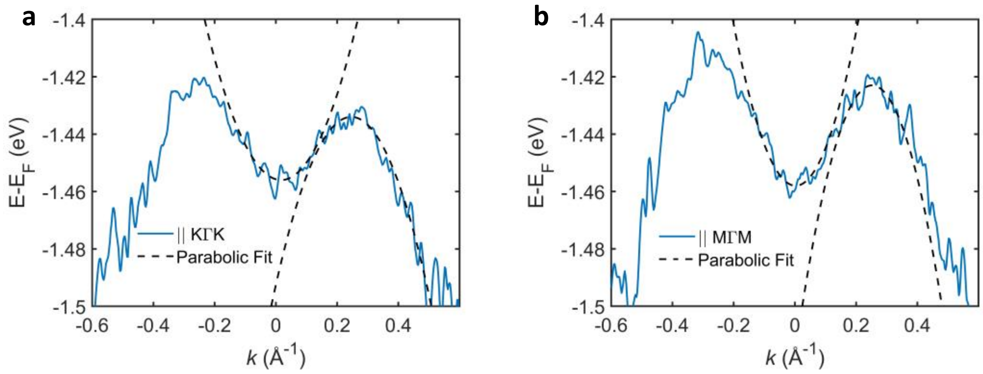

Supplementary Figure 8. Parabolic fits to energy dispersions

Experimental dispersion of the uppermost valence band of β-In_2Se_3 with their parabolic fittings (dashed lines) along the (a) $\bar{\Gamma} \rightarrow \bar{K}$ and (b) $\bar{\Gamma} \rightarrow \bar{M}$ directions. The derived hole effective masses are shown in Table 2 of the manuscript.

Supplementary Note 9. DFT for single layer α-In_2Se_3 and β-In_2Se_3

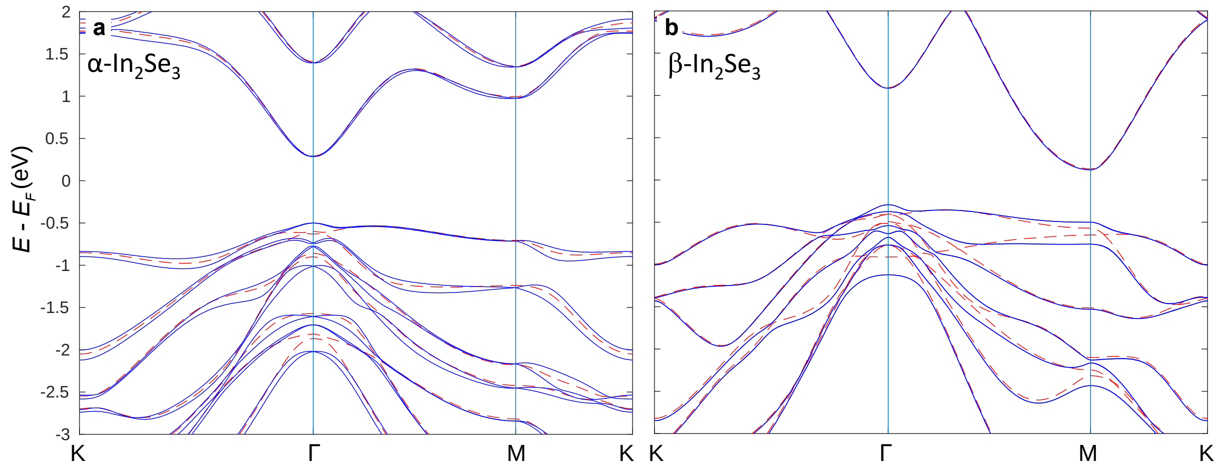

Supplementary Figure 9. Band structure for single layer α-In_2Se_3 and β-In_2Se_3

Calculated band structures for single layer α-In_2Se_3 (left) and β-In_2Se_3 (right). Blue curves include SOC, red dashed curves are without SOC.

References

1. Liu, L. *et al*. Atomically Resolving Polymorphs and Crystal Structures of In_2Se_3. *Chem. Mater*. **31**, 10143 (2019).
2. Balakrishnan, N. *et al*. Quantum confinement and photoresponsivity of β-In_2Se_3 nanosheets grown by physical vapour transport. *2D Materials* **3**, 025030 (2016).
3. O'Donnell, K. & Chen, X. Temperature dependence of semiconductor band gaps. *Applied Physics Letters* **58**, 2924-2926 (1991).
