# Supplementary material for: Probing and manipulating the Mexican hat-shaped valence band of In2Se3
Source: Nat Commun. 2025 Jan 22;16:922. doi: 10.1038/s41467-025-56139-8 (PMC11754478; doi:10.1038/s41467-025-56139-8)
Supplement: Supplementary file 2 — Description of Additional Supplementary Files [file 41467_2025_56139_MOESM2_ESM.docx]

Description of Additional Supplementary Files

**File Name:** Supplementary Movie 1

**Description:** Color plot of the electron energy (in eV) versus in-plane k-vector for different out-ofplane wave vectors (α-In2Se3).
