# Supplementary figures and images for: Probing and manipulating the Mexican hat-shaped valence band of In2Se3
Source: Nat Commun. 2025 Jan 22;16:922. doi: 10.1038/s41467-025-56139-8 (PMC11754478; doi:10.1038/s41467-025-56139-8)

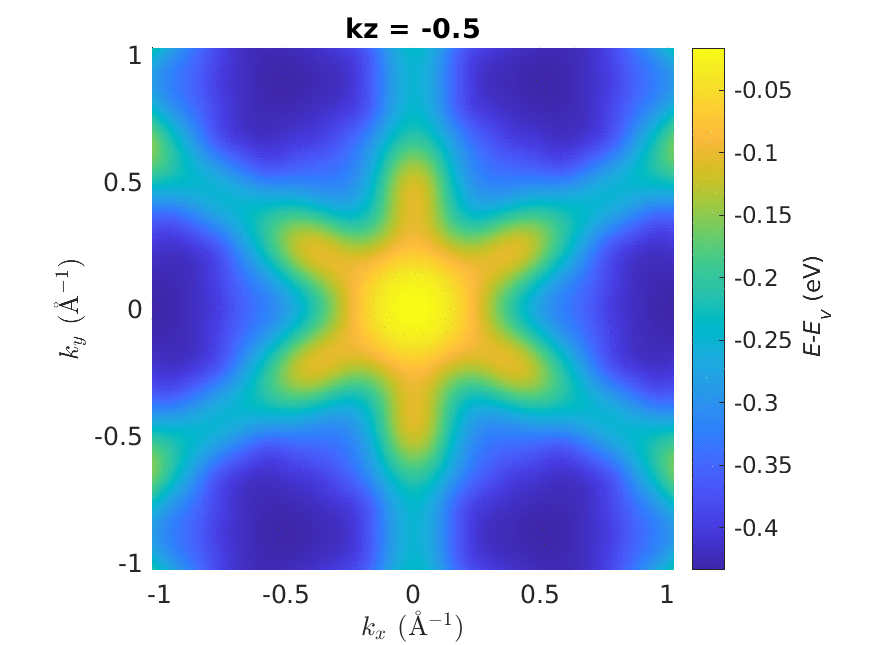

Supplement: Supplementary file 3 — Supplementary Movie 1 [file 41467_2025_56139_MOESM3_ESM.gif]
